# Understanding and measuring quality of care: dealing with complexity

**DOI:** 10.2471/BLT.16.179309

**Published:** 2017-03-20

**Authors:** Johanna Hanefeld, Timothy Powell-Jackson, Dina Balabanova

**Affiliations:** a London School of Hygiene & Tropical Medicine, Keppel Street, London, WC1E 7HT, England.

## Abstract

Existing definitions and measurement approaches of quality of health care often fail to address the complexities involved in understanding quality of care. It is perceptions of quality, rather than clinical indicators of quality, that drive service utilization and are essential to increasing demand. Here we reflect on the nature of quality, how perceptions of quality influence health systems and what such perceptions indicate about measurement of quality within health systems. We discuss six specific challenges related to the conceptualization and measurement of the quality of care: perceived quality as a driver of service utilization; quality as a concept shaped over time through experience; responsiveness as a key attribute of quality; the role of management and other so-called upstream factors; quality as a social construct co-produced by families, individuals, networks and providers; and the implications of our observations for measurement. Within the communities and societies where care is provided, quality of care cannot be understood outside social norms, relationships, trust and values. We need to improve not only technical quality but also acceptability, responsiveness and levels of patient–provider trust. Measurement approaches need to be reconsidered. An improved understanding of all the attributes of quality in health systems and their interrelationships could support the expansion of access to essential health interventions.

## Résumé

Les définitions et approches d'évaluation existantes de la qualité des soins de santé ne couvrent souvent pas toutes les complexités en jeu pour correctement appréhender la qualité des soins. Ce sont les perceptions de la qualité, plutôt que les indicateurs cliniques de la qualité, qui expliquent généralement le recours aux services de soins et déterminent l'augmentation de la demande. Dans cet article, nous nous penchons sur la nature de la qualité, sur la manière dont les perceptions de la qualité influent sur les systèmes de santé et sur ce que ces perceptions indiquent en termes d'évaluation de la qualité au sein des systèmes de santé. Nous y évoquons six défis spécifiques, liés à la conceptualisation et à l'évaluation de la qualité des soins: la qualité perçue en tant que facteur déterminant de l'utilisation des services; la qualité en tant que concept façonné au fil du temps par l'expérience; la réactivité en tant que caractéristique clé de la qualité; le rôle des pratiques de gestion et d'autres facteurs « en amont »; la qualité en tant que construction sociale coproduite par les familles, les individus, les réseaux et les prestataires; et les implications de nos observations en termes d'évaluation. Dans les communautés et sociétés dans lesquelles les soins sont dispensés, la qualité des soins ne peut pas être comprise indépendamment des normes, relations et valeurs sociales et du climat de confiance en présence. Nous devons améliorer non seulement la qualité technique mais aussi l'acceptabilité, la réactivité et le climat de confiance entre les patients et les prestataires de soins. Il est donc nécessaire de reconsidérer les approches d'évaluation. Une meilleure compréhension de toutes les facettes de la qualité dans les systèmes de santé et de leurs corrélations pourrait contribuer à étendre l'accès aux interventions sanitaires essentielles.

## Resumen

Las definiciones y los enfoques de medición existentes de la calidad de la atención sanitaria no suelen abordar las complejidades involucradas en la comprensión de la calidad de la atención. Son las percepciones de la calidad, y no los indicadores clínicos de calidad, lo que impulsa la utilización de los servicios y son esenciales para el aumento de la demanda. Este artículo se centra en la naturaleza de la calidad, la forma en que las percepciones de la calidad influencian los sistemas sanitarios y qué indican dichas percepciones sobre la medición de la calidad de los sistemas sanitarios. Se analizan seis desafíos específicos relacionados con la conceptualizacion y la medición de la calidad de la atención: la calidad aparente como un impulsor de la utilización de los servicios; la calidad como un concepto formado con el tiempo a través de la experiencia; la capacidad de respuesta como un atributo fundamental de la calidad; el papel de la gestión y otros factores denominados previos; la calidad como una construcción social coproducida por las familias, individuos, redes y profesionales; y las implicaciones de nuestras observaciones para la medición. Dentro de las comunidades y sociedades en las que se ofrece atención, la calidad de la misma no puede concebirse sin tener en cuenta las normas sociales, las relaciones, la confianza y los valores. Es necesario mejorar la calidad técnica, así como la aceptación, la capacidad de respuesta y los niveles de confianza entre paciente y profesional. Conviene reconsiderar los enfoques de medición. Una mejor comprensión de todos los atributos de la calidad de los sistemas sanitarios y sus interrelaciones podría dar apoyo a la expansión del acceso a intervenciones sanitarias básicas.

## ملخص

تعجز التعريفات والأساليب المنهجية للقياس القائمة حاليًا والخاصة بجودة الرعاية الصحية عن علاج مشكلة التعقيدات التي تشوب استيعاب جودة الرعاية، فالتصورات السائدة عن الجودة – وليست المؤشرات السريرية لقياسها – هي الكفيلة بالتشجيع على الاستفادة من الخدمات والضرورية لزيادة حجم الطلب عليها. تهدف هذه الدراسة إلى مراجعة طبيعة الجودة، وتأثير التصورات السائدة على الأنظمة الصحية، ومدلول تلك التصورات بالنسبة لقياس الجودة في إطار الأنظمة الصحية. إننا نناقش ستة تحديات معينة تتعلق بصياغة المفاهيم حول جودة الرعاية وقياسها، والتي تتمثل في: الجودة الملحوظة باعتبارها دافعًا للاستفادة من الخدمات؛ والجودة كمفهوم يتشكل بمرور الوقت من واقع خبرة التعامل؛ والاستجابة باعتبارها ميزة أساسية للجودة؛ ودور الإدارة وغيرها مما يعرف باسم العوامل المعيقة؛ والجودة باعتبارها نمط اجتماعي يعود منشأه إلى الأسرة والفرد والشبكات ومقدمي الرعاية، وآثار ملاحظاتنا بالنسبة للقياس. وفي الأوساط والمجتمعات التي يتم تقديم الرعاية فيها، لا يمكن فهم الجودة خارج إطار العلاقات والثقة والقيم والمعايير الاجتماعية. لسنا بحاجة إلى تطوير الجودة الفنية وحسب، وإنما العمل أيضًا على تحسين عناصر القبول والاستجابة ومستوى الثقة المتبادلة بين المريض ومقدم الرعاية. وهناك حاجة لإعادة النظر في الأساليب المنهجية للقياس، لأن تطوير فهم جميع سمات الجودة في الأنظمة الصحية والعلاقات المتبادلة فيما بينها كفيل بتعزيز الجهود الرامية لتيسير سبل الوصول إلى خدمات التدخلات الصحية الأساسية.

## 摘要

在了解护理质量的过程中，现有的医疗保健质量定义和衡量方法往往无法解决所涉及的复杂问题。 这是对质量的看法，而不是临床质量指标，它推动着服务利用率并且对不断增长的需求至关重要。 我们在这里反思质量的本质、对质量的看法如何影响卫生系统，以及此类看法表明卫生系统内的质量衡量具有什么意义。 我们讨论与护理质量的概念化和衡量相关的六个具体挑战： 将质量视为服务利用率的推动因素；将质量视作通过长期积累的经验形成的概念；将响应性视作质量的关键属性；管理以及其他所谓的上游因素的作用；将质量视作由家庭、个人、网络和医疗机构共同构造的社会建构；以及我们观察衡量过程的意义。 在提供护理的社区和社会中，无法脱离社会规范、关系、信任和价值观了解护理质量。 我们不仅需要改进技术质量，还需要提高可接受性、响应性以及患者和医疗机构之间的信任程度。 需要重新考虑衡量方法。 更好地了解卫生系统质量的所有属性及其相互关系有助于扩大基本卫生干预措施的使用范围。

## Резюме

Существующие определения и подходы к измерению качества медицинского обслуживания часто не учитывают сложности, связанные с пониманием качества медицинской помощи. Именно субъективная оценка (а не строго объективные показатели качества) побуждает использовать услуги и имеет важное значение для повышения спроса. В данной статье мы рассмотрим основные свойства качества и то, как субъективная оценка качества влияет на системы здравоохранения и что такие субъективные оценки говорят об измерении качества в системах здравоохранения. Мы обсудим шесть конкретных задач, связанных с концептуализацией и измерением качества медицинской помощи: воспринимаемое качество как определяющий фактор использования услуг; качество как концепция, сформированная с течением времени на основе опыта; ответная реакция как ключевой атрибут качества; роль руководства и других вышестоящих инстанций; качество как социальное понятие, совместно определяемое семьями, отдельными лицами, сетями и медицинскими учреждениями; а также значение наших наблюдений для измерения качества. В сообществах и странах, где оказывается медицинская помощь, качество помощи необходимо рассматривать в контексте социальных норм, отношений, доверия и ценностей. Нам необходимо улучшить не только техническое качество, но и приемлемость, ответную реакцию и уровень доверия между пациентом и врачом. Необходимо пересмотреть методы измерения. Улучшение понимания всех атрибутов качества в системах здравоохранения и их взаимосвязей может способствовать расширению доступа к основной медицинской помощи.

## Introduction

Policies to improve population health have often focused exclusively on the expansion of access to basic health services, to the neglect of quality of care. Efforts to increase the demand for priority interventions have implicitly assumed that the care available is of sufficient quality or that, with the expansion of coverage, quality will naturally improve.^1^ However, such assumptions may be incorrect. There is growing recognition that people may be acting in a perfectly rational way when they avoid using health services of poor quality and that poor quality of care can be a barrier to universal health coverage independent of access.^2^

The aim of many strategies to improve health-care quality has been to ensure that essential inputs – e.g. technology, operational facilities, pharmaceutical supplies and trained health workers – are in place.^3^ Many such strategies have focused on the supply side and been designed to support the provision of services according to clinical guidelines.^4^ The acknowledgement that quality improvement approaches should be applied within patient-centred models of care is relatively recent.^5^

In this paper we seek to unpack complexities around quality of care and identify strategies for improving the measurement of such quality. An understanding of these issues could inform pragmatic strategies for the analysis and measurement of quality of care. We draw on research conducted in a variety of low- and middle-income countries and identify areas of inherent complexity that require further in-depth research. In doing so, we reflect on what is meant by quality of care and how perceptions and understanding of quality of care influence health systems and effect the measurement of quality.

We have identified and structured our discussion around six conceptual and measurement challenges. First is the recognition that, even though they may not reflect actual quality, perceptions of the quality of care are an important driver of care utilization. Second, a patient’s experience of quality must be conceptualized as occurring over time. Third, responsiveness to the patient is a key attribute of quality. Fourth, so-called upstream factors – e.g. management at facility and higher levels – are likely to be important for quality. Fifth, quality can be considered as a social construct co-produced by different actors. Finally, there are substantial measurement challenges that require the adaptation and improvement of current approaches.

The classic framework on quality of care developed by Donabedian makes the distinction between structure, process and outcomes.^6^ More recently, the Institute of Medicine in the United States of America (USA) has unpacked the concept further and suggested that efforts to improve care quality should be focused around six aims: effectiveness, efficiency, equity, patient-centredness, safety and timeliness. We do not seek to propose a new framework for understanding quality. Rather, we highlight some key issues that deserve more consideration in debates about enhancing the accessibility and quality of care. Building on our experiences of doing empirical research in low- and middle-income countries, we present several insights that are complementary to existing, comprehensive frameworks of quality of care and may be absent from current debates.

## Clinical quality

Clinical quality of care relates to the interaction between health-care providers and patients and the ways in which inputs from the health system are transformed into health outcomes. The care provided should be effective, evidence-based and neither underused nor overused.^7^ The concept of clinical effectiveness tends to shift attention away from inputs such as drugs and equipment and towards the process of care.^6^^,^^8^ While relatively easy to measure, the availability of inputs cannot generally be used in isolation to determine if a patient’s health is likely to improve as a result of the care received.^9^ Clinical processes are directly attributable to the behaviour of health-care providers and their measurement can provide a critical starting point in the development of methods to improve care received by patients. Although health outcomes can be informative, they are only likely to be a crude measure of quality because of the inherent unpredictability in patients’ responses to health care.^9^

Assessment of the clinical quality of care poses several conceptual and practical challenges. It requires a strong evidence base that can act as a benchmark against which to evaluate interventions. In high-income countries, treatments received can be compared with the treatments recommended in national guidelines. In many low- and middle-income countries, however, such guidelines are either not available or poorly enforced. Even when such guidelines are present, the evaluation of what constitutes the overprovision of care is not clear-cut and requires careful judgement. Although harmful care should be distinguished from unnecessary care, such categorization can be difficult in practice. Care for a single patient may be provided over the course of numerous interactions by a large team of health professionals. In such circumstances, measurement of the quality of care often focuses on a small number of distinct interventions with proven efficacy.

There are several well-known practical challenges to the assessment of the clinical quality of care. For example, it may not be possible to observe the interactions between patients and their physicians and, when they are possible, such observations can generate bias through the Hawthorne effect, i.e. health-care providers change their behaviour when observed.^10^ In low- and middle-income countries, medical records are often poorly maintained and may not reflect actual practice. The use of so-called undercover or standardized patients in the assessment of clinical care may raise ethical concerns,^11^ is generally limited to non-invasive conditions^12^ and is not a practical solution to the routine measurement of quality.^9^ Despite these challenges, an influential literature on the clinical quality of care in low- and middle-income countries is emerging.^2^^,^^13^

## Perceived quality

Attempts to improve the quality of care have often been underpinned by a biomedical understanding of quality – i.e. the conceptualization of a gold standard of quality guided by clinical guidelines – that can lead to a narrow focus. Provider practices tend to vary despite the existence of accountability procedures and guidelines.^14^ Interventions may not be implemented as intended or easily accommodated within established models of care.^15^ Clinical quality is important for patient outcomes but perceptions of the quality of care – which may not correlate with actual quality – are likely to be the key drivers of utilization.^16^^,^^17^ Patients may also find it difficult to evaluate the quality of care because they lack their physician’s medical expertise and training.^18^^,^^19^

In South Africa, a key motivating factor in patients’ travel to access health services – including travel across borders – was found to be the patients’ perceptions of the quality of health services.^20^ Patients may sometimes believe an ineffective and unsafe treatment to be good, even when they have access to effective and safe treatments. In Malaysia, for example, many people with hypertension seek potentially ineffective and unsafe treatments from traditional practitioners.^21^ Perceptions of the quality of care are based on a mix of individual experience, processed information and rumour. In Uganda, perceptions of the quality of the care that was locally available were found to have persuaded many women to seek maternal care away from their local area – apparently regardless of the availability of transportation and the distances involved.^22^ In Bangladesh, despite a nationwide expansion in the network of health facilities, facility-based deliveries remained rare and most women still attempted to give birth at home or, in the case of complications, at distant periurban health centres that the women believed to offer care of higher quality than that available at the community facilities closest to their homes.^23^^,^^24^ Patients’ trust in services has been shown to be an important element of perceived quality.^25^

Perceptions of the quality of care may relate entirely to non-clinical factors. For example, criminalized or marginalized populations – e.g. some ethnic or sexual minorities – may judge the quality of care only according to the extent that the care environment is non-discriminatory or supportive.^26^ In Zambia, many patients considered public-sector clinics supported by one particular nongovernmental organization to be better than other public-sector facilities that apparently provided the same standardized package of care.^27^

The effect of perceived quality is not limited to delivery models. Among remote rural populations in Armenia, there was disappointingly low participation in community-based health-insurance schemes because the quality of the care provided by the schemes was perceived to be low. Despite the often high out-of-pocket costs, most people in the communities covered by the schemes preferred to use district-based clinics and hospitals – where they believed the quality of care to be higher than in the facilities covered by the schemes.^28^ Although quality is a construct largely based on individual subjective perceptions, such perceptions are shaped by collective and traditional beliefs and peer influences. While improving or, at least, maintaining the actual quality of the care they provide, health systems need to address – and ultimately close – the gap between perceived and actual quality.

## Quality as a process

There is a temporal dimension to both clinical and perceived quality. Although the Donabedian framework recognizes the importance of understanding the process of care,^6^^,^^8^ the quality of care may often be assessed in just a single encounter or illness episode. However, individual treatment for most diseases is not a one-off event but a succession of treatment episodes. Patients’ perceptions of quality may develop over time, as the different attributes of the services available and their outcomes are revealed. Waiting times and staff attitudes may be perceived rapidly. However the patient’s experience of clinical treatment, e.g. surgery, and its implications for subsequent care, e.g. frequent check-ups, and health outcomes, e.g. potential complications, may carry on developing over months or years. Patients may only become sensitized to the benefits of having a dedicated provider and effective follow-up after they experience the absence of such benefits. Easy-to-navigate pathways to care and continuity are critical to how patients perceive the quality of care and choose whether to continue treatment or not.^29^ Long-term compliance is only likely if the patients involved consider their care to be of good quality. Such compliance is a particular challenge in the monitoring and treatment of chronic noncommunicable diseases and human immunodeficiency virus, especially for the under-resourced health systems of low- and middle-income countries.^30^^–^^33^

## Responsiveness

While *The World health report 2000. Health systems: improving performance*^34^ defined responsiveness to people’s non-medical expectations as a key health-systems goal, the relationship between responsiveness and quality has rarely been discussed. Although ability to book an appointment, confidentiality, privacy, respect shown by staff and waiting times are not service attributes that are clinically necessary, they may all influence patients’ perceptions and their willingness to return for – or adhere to – treatment. At a broader level, responsiveness involves respect for cultural needs and the preferences of specific patient groups – e.g. ethnic, gender and sexual minorities and migrants. The relationship between health workers and their patients often develops over time and multiple episodes of care. As levels of trust and mutual understanding increase, responsiveness and the patients’ perceptions of the quality of their care often improve.^35^

Although responsiveness to need is often consistent with good clinical practice, it represents an added layer in the patients’ perceptions of quality. In one South African study, women appeared to have been given greater access to public maternity wards but it was the verbal abuse that the women often suffered on such wards that largely shaped the women’s poor perceptions of the care that they had received.^36^

## Upstream factors

The patient–provider interaction is likely to be influenced by governance and management practices at national, subnational and facility levels. The results of studies in the United Kingdom of Great Britain and Northern Ireland and the USA have demonstrated the key importance of management in ensuring care of high quality.^37^ In low- and middle-income countries, however, there appears to have been little consideration of the role of management practices – especially at district or facility level – in influencing the quality of care. There is increasing recognition that health professionals do not act in isolation and that governance, management and structural factors also determine the performance of health systems.^38^^,^^39^

Even when frontline providers do have substantial discretion in their interpretation of regulations and freedom to adapt treatment protocols, their actions may still largely depend on upstream factors related to institutional capacity, legal sanctions and professional norms. A study of tuberculosis cases in Samara, in the Russian Federation, revealed that while entry to the care system was relatively easy and formally free and pharmaceuticals were highly subsidized, some cases from marginalized groups – e.g. former prisoners, migrants and people not registered with the authorities – still avoided treatment because of perceived discrimination, loss of social status and stigma.^40^ Both behavioural and structural factors can be important when assessing perceived quality of care.

## Quality as a social construct

Assessment of quality of care in low- and middle-income countries is frequently conducted at the individual level by using various tools – e.g. clinical observations, exit and in-depth interviews, extraction of medical records, role-playing vignettes and standardized patients, designed to assess both patients’ experiences and technical quality. However, social networks influence perceptions relating to both health services and illness.^41^ Therefore, for a comprehensive investigation of the development of the general public’s and patients’ perceptions of the quality of care, we need to examine community and family values.

In many situations, patients may have responses to a health provider’s actions and, similarly, providers may adapt their responses to patients to suit social norms.^42^ For example, a patient may be recommended a clinical investigation and they may either agree to be investigated – e.g. if the proposed investigation is offered by a provider trusted by the patient’s social network – or they may exit the system and seek care elsewhere, e.g. from a more trusted traditional practitioner. Such responses may be considered as a social relationship that can happen in formal care settings, or elsewhere.

Perception of quality can also be shaped by power relationships in society. In a study in the Russian Federation, the women most likely to undergo pregnancy-related procedures were found to be the relatively young and poorly educated. Although such women were relatively poor and therefore found it particularly hard to pay for their care, they appeared to be given little choice – possibly because of their relatively low social status and inability to negotiate care that was commensurate to their needs.^43^ Similar discrepancies between what health professionals felt would improve the quality of care for non-compliant patients and those patients’ preferences and wishes were observed in a study of tuberculosis cases in India. In that study, the number of treatment choices offered was found to be positively correlated with social status.^44^

## Measurement challenges

In light of the above discussion, there is a case for taking a broader perspective when measuring quality of care. Although this has been recognized by the World Health Organization’s monitoring framework for universal health coverage^45^ – which considers effectiveness of treatment, patient safety, people-centredness and the level of integration of health services as key dimensions – the focus of recent assessments of the quality of care has been on indicators of health-service coverage.^45^^,^^46^

We suggest that, for a comprehensive and detailed assessment of the quality of health services, both clinical and perceived quality of care need to be evaluated and then compared (Box 1). Alongside technical measures of quality, attention should be given to manifestations of quality – e.g. acceptability, cultural appropriateness and responsiveness. Strategies to improve clinical quality only have the potential to increase demand for care if the general public’s perceptions of the quality of the care available also improve.

Box 1 **Principles for measuring the quality of health care**

- Measure aspects of care that go beyond technical quality, e.g. responsiveness, acceptability and trust.
- Measure perceived quality and compare with clinical quality.
- Measure quality at different points in the patient pathway through the health system.
- Measure the immediate and upstream drivers of quality of care.
- Measure collective and individually assessed quality and its relationship to power, social norms, trust and values.

Any evaluation of the overall quality of care needs to consider a patient’s experience of quality as a cumulative process. Changing patterns of illness and increasing numbers of treatment options mean that an increasing amount of health care involves a sequence of interlinked contacts – with a range of health professionals at different levels of the health system – over a lengthy period.^47^ A patient’s perceptions may vary widely as treatment follows diagnosis and follow-up follows treatment, with each stage potentially affecting the patient’s subsequent choices. By measuring clinical and perceived quality at each key step in this continuum of care, it should be possible to generate a better, more nuanced understanding of how patients interact with health systems.

A growing body of work focusing on measures of patients’ perceptions now exists. To understand these perceptions more holistically, qualitative methods need to become an integral part of quality assessments. In such assessments, theory-driven hierarchical models can be useful in generating propositions to guide empirical research or help deepen interpretation.^48^ Mid-range programme theories^48^ and open-box evaluations^49^ have also been useful in examining why and how particular health programmes work. Although the measurement of indicators that are rapidly observed by patients seeking care – e.g. staff attitudes and waiting times – can be useful, it is important to delve deeper and study how upstream factors, such as management practices, matter – e.g. by influencing staff morale. Use of carefully selected proxies for quality of care and comparison of findings generated through different methods may help to inform pragmatic intervention strategies.

Finally, assessment of individual perceptions of the quality of care and examination of how such perceptions are rooted in community, family and societal expectations, norms and values may offer a promising way forward. Perceived quality may correlate closely with the expectations and social status of the users themselves, the circumstances in which the users obtain care and/or the levels of community cohesion and resources that enable collective action. Although the inclusion of contextual variables and appropriate units of observation for studying community and social group-level characteristics may be methodologically challenging, it is important for understanding individual choices and perceptions.

## Conclusion

Recognition of the multifaceted nature of the quality of care is critical for scaling up priority health interventions. If uptake of health services is to be increased, we require not only better technical quality but also better acceptability and patient-centredness – across the continuum of care. Perceptions of quality are shaped by interconnected community, health-system and individual factors. Moreover, quality of care cannot be understood fully without some appreciation of the social norms, relationships and values and trust within the communities and societies where care is provided.
